# Outcomes of Complex Iatrogenic Biliary Tract Injuries: Retrospective Single-Center Experience from Pakistan

**DOI:** 10.12669/pjms.42.3.14851

**Published:** 2026-03

**Authors:** Nadir Farid, Muhammad Yasir Khan, Ihsan Ul Haq, Sohail Rashid

**Affiliations:** 1 Nadir Farid, FCPS, Department of HPB & Liver Transplant, Pakistan Kidney and Liver Institute and Research Center, Lahore Pakistan; 2 Muhammad Yasir Khan, FCPS, Department of HPB & Liver Transplant, Pakistan Kidney and Liver Institute and Research Center, Lahore Pakistan; 3 Ihsan Ul Haq, FCPS, Department of HPB & Liver Transplant, Pakistan Kidney and Liver Institute and Research Center, Lahore Pakistan; 4 Sohail Rashid, FCPS, Department of HPB & Liver Transplant, Pakistan Kidney and Liver Institute and Research Center, Lahore Pakistan

**Keywords:** Iatrogenic bile duct injury, Bile duct repair, Biliovascular injury, Hepaticojejunostomy

## Abstract

**Background & objective:**

Iatrogenic bile duct injury (IBDI) is a well-established complication of cholecystectomy. Effective management of this complex surgical complication necessitates early referral to high-volume hepatobiliary centers. The sequelae of this injury can lead to biliary peritonitis, recurrent cholangitis, biliary stricture, liver cirrhosis, sepsis, and even mortality. The aim of this study was to thoroughly examine the clinical presentation, injury grading, surgical approaches, and outcomes of patients with IBDI.

**Methodology:**

Data of all IBDI patients were collected, and 79 patients were included who underwent surgery between 1st January 2019 to 31st January 2025 at Hepato pancreato biliary department Pakistan Kidney and Liver Institute and Research Centre. Our primary outcomes included the success rate of different surgical procedures, postoperative morbidity and mortality, need for hepatic resection or liver transplantation, and length of hospital stay. Categorical data such as age, gender, risk factors, and diagnosis were presented as frequencies and percentages. Crude odds ratios (ORs) with 95% confidence intervals were determined for the risk of death.

**Results:**

A total of 79 patients met the selection criteria, including 18 (22.8%) males and 61 (77.2%) females. The median age of the patients was 43 years (IQR, 34-53 years). The majority of IBDI cases, 48 (60.8%), were treated with hepaticojejunostomy, followed by 16 (20.3%) with redo hepaticojejunostomy, and one patient underwent liver transplantation for secondary biliary cirrhosis. Right hepatectomy with biliary reconstruction was performed in 45.4% of cases with isolated RHA injury. The overall success rate was 96.2%, irrespective of the type of surgery performed. The median follow-up duration for the 77 patients was 20 months (IQR, 11-31 months). Two (2.5%) patients were lost to follow-up after the initial visit, and hospital mortality was observed in 2 (2.5%) patients. There was no statistically significant difference in postoperative complications across the different risk factors or surgical types. Postoperative morbidity occurred in 23 patients (29.1%), and these complications were more pronounced in E5 injuries associated with vascular involvement.

**Conclusion:**

IBDI represents a complex surgical complication that demands expertise, and inadequate reconstruction by the index surgeon should not be attempted. Early referral to high-volume hepatobiliary centers is recommended. Complex biliary and vascular injury is an independent risk factor for hepatectomy and significantly prolongs postoperative recovery but does not substantially compromise the long-term outcomes when adequately treated.

## INTRODUCTION

Iatrogenic bile duct injury (IBDI) is a well-recognized but preventable complication of cholecystectomy.^1^,^2^ The sequelae of this injury may include biliary peritonitis, stricture, hepatolithiasis, secondary biliary cirrhosis, sepsis, and even death.^3^ The incidence of major bile duct injuries after cholecystectomy ranges from 0.08% to 0.3%,^4^ while that of minor injuries ranges from 0.3% to 1.5%.^5^ Although uncommon, the case fatality rate of IBDI is reported to be 3-5.7%^5^ and results in significant clinical and economic burden on patients as well as the healthcare system.^6^

Immediate surgical repair can result in good outcomes at specialized centers; however, the success rate for primary surgeons performing repair is only 17-30%. Therefore, timely recognition and early referral are highly recommended.^7^,^8^ Meta-analyses have shown that complex biliovascular injury and repair performed outside specialized centers are independent predictors of treatment failure and worse outcomes.^9^ Definitive surgery should be delayed until the patient is hemodynamically stable and the infections have resolved, which may take several months.^10^

Risk factors for IBDI include anatomical variations, cholecystitis, severe obesity, previous surgery, underlying liver disease, unexpected bleeding, surgeon’s inexperience, and excessive dissection of Calot’s triangle.^11^ Various surgical techniques have been introduced to prevent biliary tract injuries, including the Critical View of Safety (CVS) method, subtotal cholecystectomy, use of anatomical landmarks, B-SAFE method, intraoperative cholangiography, and near-infrared fluorescent cholangiography.^1^

There are limited data on the management of iatrogenic biliovascular injuries in this resource-limited region. This study was conducted to emphasize the importance of centralized registries or referral helplines, as delayed referrals are associated with increased morbidity and mortality.

## METHODOLOGY

In this retrospective single-center cohort study, total of 79 patients were enrolled based on the availability and completeness of data in the hospital records. We divided our patients into two groups. Primary referred (Group-A) patients were those who had IBDI but did not undergo any therapeutic intervention at the referring hospital, while secondary referred (Group-B) patients underwent surgical, endoscopic, or radiologic interventions. The study was conducted at the Department of Hepato-Pancreato-Biliary and Liver Transplant Unit after approval from the Institutional Review Board (IRB) of Pakistan Kidney and Liver Institute and Research Centre (PKLI & RC), Lahore, Pakistan. The study was carried out by reviewing medical records from January 1, 2019 to January 31, 2025. A non-probability consecutive sampling technique was used to include all eligible cases All patients referred to our HPB department who had iatrogenic bile duct injury during cholecystectomy of any age and gender were included in this study. The excluded patients were those with missing or unavailable records, Minor leaks which were managed conservatively and Biliary injuries either through trauma or procedures like ERCP, Biliary tract or gastrointestinal malignancy.

### Ethical Approval:

The study was approved by the Institutional board review of Pakistan Kidney and Liver Institute and research center, Lahore (PKLI-IRB/AP/214; Dated: July 3rd 2014).

The data were collected from Sisoft Healthcare Information Systems. This database contains comprehensive clinical and pathological data for each patient..All baseline investigations were performed. MRCP or endoscopic retrograde pancreaticography(ERCP) was done after optimization where indicated. All patients were medically optimized before the intervention.Age, gender, comorbid disease, operation (laparoscopic or open cholecystectomy), injury presentation (early or late), decompression procedure (open or PTBD), investigations and radiological MRCP, type of injury according to Strasberg or Stewart way classification, associated vascular injury, postoperative morbidity and mortality, and days of hospital admission.

### Statistical Analysis:

Data were analyzed using SPSS version [version 27.0]. Continuous variables are expressed as mean ± standard deviation (SD) or median with interquartile range (IQR), depending on data distribution. Categorical variables are presented as frequencies and percentages. Comparisons between groups (such as primary vs. secondary referrals and different surgical procedures) were performed using the chi-square test or Fisher’s exact test for categorical variables and the independent-samples t-test or Mann-Whitney U test for continuous variables, as appropriate. Multivariate analysis was conducted to identify independent predictors of postoperative complications. Changes in bilirubin levels over time and between groups were assessed using repeated measures ANOVA. A p-value < 0.05 was considered statistically significant.

## RESULTS

In this study, 79 patients met the selection criteria: 18 (22.8%) were males and 61 (77.2%) were females. The median age of the patients was 43 years (IQR, 34-53 years). Among them, 32 (40.5%) were primary and 47 (59.5%) were secondary referred patients. Most patients had undergone open cholecystectomy (41, 51.9%) compared to laparoscopic procedures (38, 48.1%) (Table-I).

**Table-I:** Frequency distribution of different risk factors.

Bassline Info	Categories	Percentage
Age Group	≤45	45(57.0%)
	>45	34(43.0%)
Gender	Male	18(22.8%)
	Female	61(77.2%)
Diagnosis	Cholelithiasis(biliary colic)	75(94.9%)
	Calculous cholecystitis	1(1.3%)
	Secondary biliary cirrhosis	1(1.3%)
	Mirizzi syndrome	2(2.5%)
Vascular Injury	Yes	14(17.7%)
	No	65(82.3%)
ERCP Attempted		31(39.2%)
Surgical Drain		9(11.4%)
Percutaneous Transhepatic Biliary Drainage (PTBD)		13(16.5%)
ERCP stent		7(8.9%)
Strasberg	E1	7(8.9%)
	E2	20(25.3%)
	E3	32(40.5%)
	E4	7(8.9%)
	E5	13(16.5%)
Surgery Type	Open	41(51.9%)
	Laparoscopic	38(48.1%)
Hepatico-jejunostomy		48(60.8%)
Redo Hepatico-jejunostomy		16(20.3%)
Hepatico-jejunostomy with Right hepatectomy		6(7.6%)
Redo-Hepatico-jejunostomy with Right hepatectomy		3(3.8%)
Hepatico-jejunostomy with Segmental Resection		3(3.8%)
Redo-Hepatico-jejunostomy with Segmental Resection		1(1.3%)
Right Hepatectomy		1(1.3%)
Liver Transplant		1(1.3%)

The majority of IBDI cases (48, 60.8%) were treated with hepaticojejunostomy, followed by 16 (20.3%) with redo hepaticojejunostomy, and one patient underwent liver transplantation for secondary biliary cirrhosis. Major liver resections were performed in 10 (12.6%) cases. Comorbidities were not identified as risk factors for IBDI. There was no statistically significant difference in postoperative complications according to different risk factors or surgical types on multivariate analysis (Table-II).

**Table-II:** Comparison of postoperative complications according to different risk factors and surgery type (Multivariate analysis).

Variables	Categories	Post op Complications		Total	P value
		Yes	No		
Age Group	≤45	13(56.5%)	32(57.1%)	45(57.0%)	0.960
	>45	10(43.5%)	24(42.9%)	34(43.0%)	
Gender	Male	5(21.7%)	13(23.2%)	18(22.8%)	0.887
	Female	18(78.3%)	43(76.8%)	61(77.2%)	
Comorbidity	Yes	2(8.7%)	15(26.8%)	17(21.5%)	0.058
	No	21(91.3%)	41(73.2%)	62(78.5%)	
Groups	Primary referred	9(39.1%)	23(41.1%)	32(40.5%)	0.873
	Secondary referred	14(60.9%)	33(58.9%)	47(59.5%)	
Strasberg	E1	2(8.7%)	5(8.9%)	7(8.9%)	0.331
	E2	4(17.4%)	16(28.6%)	20(25.3%)	
	E3	8(34.8%)	24(42.9%)	32(40.5%)	
	E4	2(8.7%)	5(8.9%)	7(8.9%)	
	E5	7(30.4%)	6(10.7%)	13(16.5%)	
Vascular Injury	Yes	6(26.1%)	8(14.3%)	14(17.7%)	0.225
	No	17(73.9%)	48(85.7%)	65(82.3%)	
Surgery at PKLI	Hepatico-jejunostomy	13(56.5%)	35(62.5%)	48(60.8%)	0.348
	Redo Hepatico-jejunostomy	3(13.0%)	13(23.2%)	16(20.3%)	
	Hepatico-jejunostomy with Right hepatectomy	3(13.0%)	3(5.4%)	6(7.6%)	
	Redo Hepatico-jejunostomy with Right hepatectomy	2(8.7%)	1(1.8%)	3(3.8%)	
	Hepatico-jejunostomy with Segmental Resection	1(4.3%)	2(3.6%)	3(3.8%)	
	Redo Hepatico-jejunostomy with Segmental Resection	1(4.3%)	-	1(1.3%)	
	Right Hepatectomy	-	1(1.8%)	1(1.3%)	
	Liver Transplant	-	1(1.8%)	1(1.3%)	

**Fig.1:**
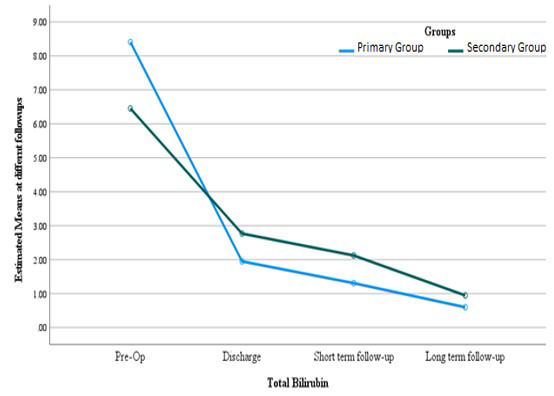
Comparison of total bilirubin at different follow ups according to group.

The mean preoperative total bilirubin level was 5.45 ± 6.63 mg/dL**,** at discharge 2.19 ± 1.98 mg/dL**,** at short-term follow-up 2.55 ± 2.37 mg/dL, and at long-term follow-up 0.54 ± 0.51 mg/dL. The difference in mean bilirubin levels between primary and secondary referred patients at each time point preoperatively (p = 0.779), at discharge (p = 0.097), at first follow-up (p = 0.286), and at long-term follow-up (p = 0.051) was not statistically significant (Fig.1). There was also no significant difference regarding bilirubin levels among different surgical groups.

The mean interval from injury to presentation was 24 months in secondary referred cases, compared to 1.6 months in primary referred cases. The overall success rate was 96.2%, irrespective of the type of surgery performed. The median follow-up duration for 77 patients was 20 months (IQR 11-31). Two (2.5%) patients were lost to follow-up after the initial visit, and hospital mortality was observed in two (2.5%) patients, both due to postoperative multiorgan failure secondary to sepsis.

Postoperative morbidity occurred in 23 (29.1%) patients and was more pronounced in E5 injuries associated with vascular injury. The hospital stay of patients who underwent redo hepaticojejunostomy with right hepatectomy or liver transplantation was significantly longer than that of other patients (p = 0.001) (Table-III).

**Table-III:** Comparison of outcome regarding surgery.

		Surgery at PKLI								
Findings		Hepatico-jejunostomy	Redo Hepatico-jejunostomy	Hepatico-jejunostomy with Right hepatectomy	Redo Hepatico-jejunostomy with Right hepatectomy	Hepatico-jejunostomy with Segmental Resection	Redo Hepatico-jejunostomy with Segmental Resection	Right Hepatectomy	Liver Transplant	
Strasberg	E1 (n=7)	7(14.6%)	-	-	-	-	-	-	-	<0.001
	E2 (n=20)	17(35.4%)	3(18.8%)	-	-	-	-	-	-	
	E3 (n=32)	19(39.6%)	11(68.8%)	2(33.3%)	-	-	-	-	-	
	E4 (n=7)	4(8.3%)	2(12.5%)	-	-	1(33.3%)	-	-	-	
	E5 (n=13)	1(2.1%)	-	4(66.7%)	3(100.0%)	2(66.7%)	1(100.0%)	1(100.0%)	1(100.0%)	
Vascular Injury	RHA (n=11)	4(100.0%)	1(100.0%)	2(66.7%)	3(100.0%)	1(50.0%)	-	-	-	0.300
	RHA and right PV injury (n=2)	-	-	1(33.3%)	-	-	-	1(100.0%)	-	
	RHA and left PV injury (n=1)	-	-	-	-	1(50.0%)	-	-	-
Vascular Injury (n=14)		4(8.3%)	1(6.3%)	3(50.0%)	3(100.0%)	2(66.7%)	-	1(100.0%)	-	<0.001
Blood Loss (>300ml) (n=28)		11(23.4%)	5(31.3%)	4(66.7%)	2(66.7%)	3(100.0%)	1(100%)	1(100%)	1(100%)	0.007
Wound Infection (n=15)		10(20.8%)	3(18.8%)	1(16.7%)	-	1(33.3%)	-	-	-	0.885
Bile Leak (n=2)		-	1(6.3%)	-	1(33.3%)	-	-	-	-	0.958
Cholangitis (n=2)		-	-	1(16.7%)	1(33.3%)	-	-	-	-	0.223
Anastomotic stricture (n=1)		1(2.1%)	-	-	-	-	-	-	-	0.995
Intra-abdominal Collection (n=3)		1(2.1%)	1(6.3%)	1(16.7%)	-	-	-	-	-	0.894
Incisional Hernia (n=3)		1(2.1%)	1(6.3%)	-	1(33.3%)	-	-	-	-	0.722
Transaminitis (n=1)				-	-	-	1(100%)	-	-	0.151
Length of hospital stay(Median, Days)		6.5(5-9)	7(6-10.75)	18(7.75-29.25)	26(7-26)	7(6-7)	6	11	25	
Duration of Follow up(Median, Months)		17(11-27)	14(7.75-28.25)	27(17.5-41.75)	29(11-29)	29(3-29)	36	36	36	

Vascular injuries associated with IBDI were observed in 14 patients (17.7 %). Isolated right hepatic artery (RHA) injury was present in 11 patients, requiring biliary reconstruction with right hepatectomy in five cases. Bismuth type E3 injury was the most prevalent, observed in 32 (40.5%) patients. Complex E5 injuries with vascular involvement required right hepatectomy with biliary reconstruction in all cases (Table-III).

## DISCUSSION

**Fig.2:**
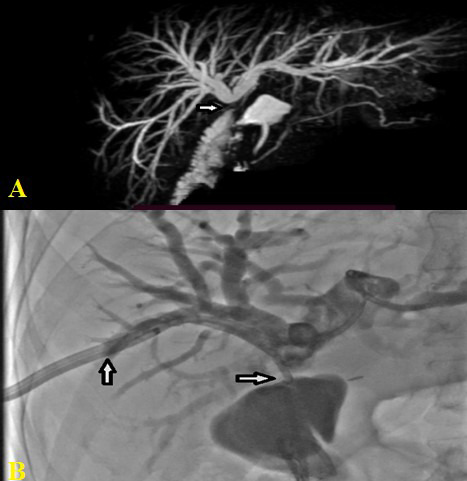
**A.** Hepaticojejunostomy stricture in a 30 years old patient (Arrow showing stricture site) **B.** PTBD stenting with upsize stenting but eventually underwent revisional surgery at our centre (arrows showing PTBD stent, traversing the stricture site).

Complex iatrogenic bile duct injury (IBDI) is a preventable yet serious complication.^1^ At our institution, we have adopted the widely accepted step-up approach, involving sepsis control, biliary drainage (via ERCP or PTBD), drainage of intra-abdominal bile collections, bilio-enteric anastomosis, liver resection, and transplantation when indicated. In our study, more than half of the patients underwent at least one intervention before referral. (Fig.2 A & B).

Early referral and identification is crucial; however, 70-80% of bile duct injuries remain unrecognized intraoperatively,^12^ which is comparable to our finding of 53.1% intraoperative recognition at referral centers. Unfortunately, in our setting, delayed referral is common because of low socioeconomic status, procedures performed at non-specialized centers, questionable surgical expertise, fear of litigation, and lack of centralized registries or referral pathways. The mean referral delay in primary referral cases was 46 days, whereas in secondary referrals it was 24 months. While Lalisang et al reported definitive referral of 19 days (range ~7-152 days.^13^

In our setup, the optimal timing of reconstructive surgeries remains controversial; however, late repair was preferred, as it provides sufficient time for medical optimization and to perform a definitive anastomosis at a well-vascularized level. Some researchers have demonstrated good outcomes when immediate repair is performed by experienced HPB surgeons under optimal conditions.^14^ In contrast to some studies the delayed repair was favoured, particularly after the failed initial repair to reduce postoperative complications.^15^

**Fig.3:**
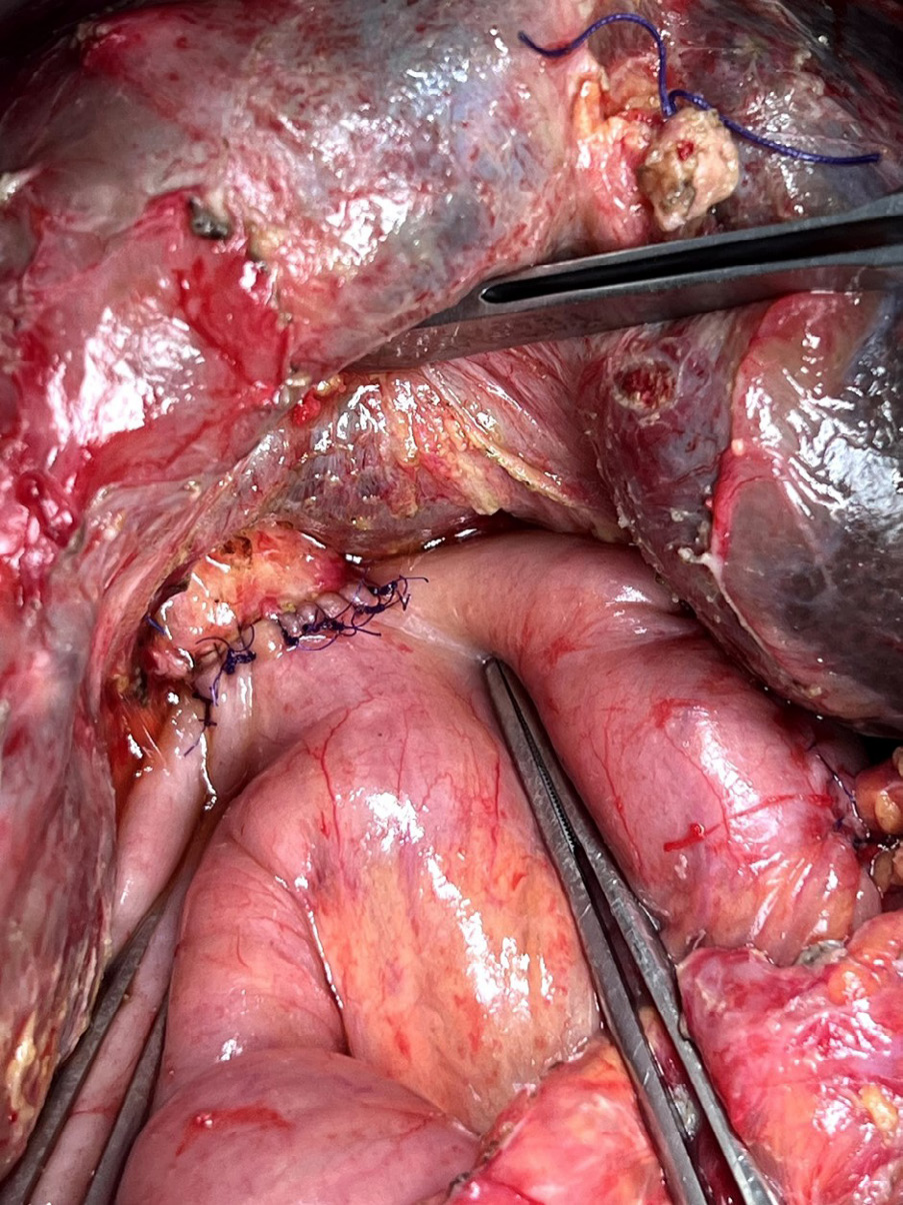
High-level biliary-enteric anastomosis using duct-to-mucosa technique.

In our practice, biliary reconstruction were carried out using duct-to-mucosa technique, aiming for high-level stricture free area and well vascularised anastomosis (Fig.3).

We observed an overall success rate of 96.2% with a median follow-up of 20 months, irrespective of the surgical approach. These findings were reported by Pesce et al., who demonstrated a 94% long-term success rate after Roux-en-Y hepaticojejunostomy.^16^ Among our patients, those who required right hepatectomy with redo hepaticojejunostomy(with left lobe of liver) experienced a longer hospital day (Median 26days) compared those who underwent redo hepaticojejunostomy alone (Median seven days). Bhat et al. reported a mean hospital stay of 10 days for redo hepaticojejunostomy alone.^17^

Our cohort demonstrated more severe biliary injuries compared to the Western literature. Consistent with published data, we found insignificant long-term results following IBDI repair both for open and laparoscopic cholecystectomy,^18^ although laparoscopic injuries were more severe.^19^ Interestingly, unlike many authors, our data showed severe injuries in open cholecystectomy, and the majority were younger patients with biliary colic. IBDI with injury to the right hepatic artery was identified in 17.7% of our cases, consistent with global estimates of 12-25%.^20^

In cases of E2-E4 strictures accompanied by isolated right hepatic artery damage, biliary reconstruction alone yielded good outcomes, whereas patients with E5 strictures and vascular injury required biliary reconstruction with right hepatectomy. The differences in the outcomes between these groups were statistically insignificant. The need for hepatectomy or liver resection was mainly due to long right-sided strictures, liver abscesses, or recurrent cholangitis.^21^ The timing of vascular repair remains debated; while some recommend delayed intervention if vascular repair is not planned, concomitant portal vein and arterial injuries may necessitate urgent hepatectomy and are associated with higher morbidity and mortality.^22^

**Fig.4:**
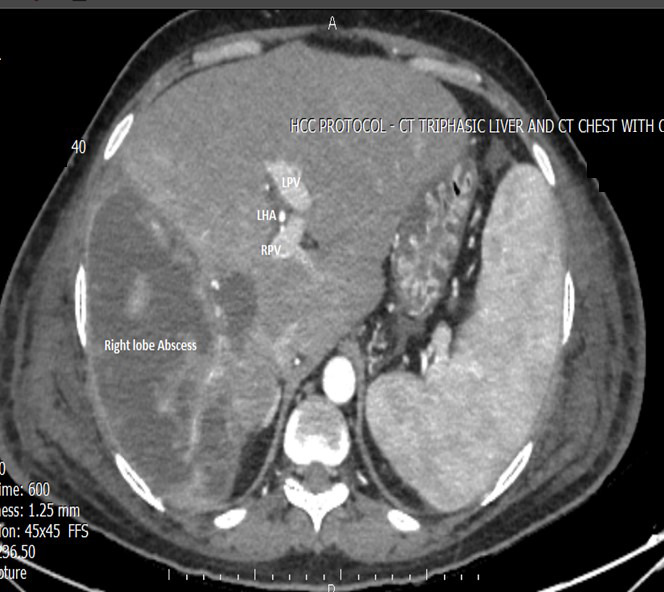
Severe hepatic ischemia following right hepatic artery and portal vein ligation.

We encountered four patients with severe hepatic ischemia secondary to ligation of the right hepatic artery and portal vein, who were referred after a mean delay of 16 days (Fig.4). Of these, two underwent surgery and two succumbed to sepsis with multiorgan failure. Among the operated patients, one died on postoperative day four after right hepatectomy due to sepsis and remnant liver failure, while the other recovered gradually despite transient small-for-size syndrome.

Major injuries involving or proximal to the biliary confluence, with or without vascular compromise, have reported short-term morbidity rates of 40-50% and mortality rates of 2-4%.^23^ The overall morbidity in our series was 29.1%. Complications were more frequent in patients with E5 injuries with vascular involvement and in those undergoing hepaticojejunostomy plus right hepatectomy (Table-III), consistent with the findings of Karvonen et al.^24^ Despite this, our overall mortality rate (2.5%) was lower than that reported in several published series (5-8 %).^25^ Biliary reconstruction carries a 5-9% risk of anastomotic failure and an 11-16% risk of stricture, most developing within 2-3 years.^26^ Two of our patients developed postoperative bile leaks, both of which required revision surgery. One patient with a complex E5 plus RHA injury later developed subacute intestinal obstruction and bile leak, which was successfully managed surgically. Another patient developed obstructive jaundice with bile leak 45 days after redo hepaticojejunostomy. The leak was initially managed percutaneously, but a third revision surgery was eventually required. Subsequently, the patient showed improvement in liver function and remained under follow-up.

Anastomotic strictures may progress to secondary biliary cirrhosis, often necessitating liver transplantation, which carries significant morbidity (50-60%) and mortality (20-30%).^27^ In our study, one 58-year-old woman developed decompensated secondary biliary cirrhosis following open cholecystectomy and hepaticojejunostomy, and subsequently underwent living-donor liver transplantation in 2021. Her postoperative course was complicated by graft perfusion abnormalities, renal dysfunction, infections, and incisional hernia, although she continues to do well with stable liver function.

Landman et al. demonstrated long-term psychological and quality of life (QoL) impairment in patients with IBDI compared to those with uncomplicated cholecystectomy.^28^ This was similarly observed in our patients, with a significant decline in both physical and psychological domains, compounded by ongoing litigation in some cases. However, long-term follow-up revealed improvements in 67 patients across both domains.

This study highlights the importance of early identification, timely referral to specialized HPB centers, and multidisciplinary management to optimize outcomes and minimize morbidity and mortality associated with IBDI. This was a single-center study highlighting the importance of early referrals, and primary surgeons were reluctant to share the details of the injury in some cases. As it is still unknown how many biliary reconstructions are performed by non-HPB specialists in the community, we are lacking a centralized IBDI registry. The results from various centers in Pakistan should also be considered. Further studies with different setups are highly recommended. Continued attention to educational programs and techniques aimed at reducing patient harm and improving surgeon skills is imperative during safe cholecystectomy.

## CONCLUSION

IBDI represents a complex surgical complication that demands expertise, and inadequate reconstruction by the index surgeon should not be attempted. Early referral to high-volume hepatobiliary centers is recommended. Complex biliary and vascular injury is an independent risk factor for hepatectomy and significantly prolongs postoperative recovery but does not substantially compromise the long-term outcomes when adequately treated.

### Authors’ Contribution:

**NF:** Conceived, data collection, designed, and editing of manuscript, is responsible for integrity of research.

**MYK, IUL and SR:** Did Drafting, Critical revision and final approval.
